# Supplementary material for: Strain diversity drives heterogeneous responses to tuberculosis combination therapy
Source: Antimicrob Agents Chemother. 2026 May 15;70(6):e01849-25. doi: 10.1128/aac.01849-25 (PMC13182990; doi:10.1128/aac.01849-25)
Supplement: Supplemental material — Supplemental methods; Tables S1 to S4; Fig. S1 to S6. [file aac.01849-25-s0001.pdf]

**Supplementary Material**

**Supplementary Methods**

Antibiotics preparation and dispensing

Each antibiotic was diluted in dimethyl sulfoxide (DMSO) to create a stock solution, which was then prepared into single-use aliquots and stored at -20°C until use. Dispensing errors were avoided with the use of the HP D300e digital dispenser to dispense drugs and drug combinations into 384-well plates, and well positions were randomized to control for plate effects.

*In vitro* model acclimation

Mtb were acclimated to cholesterol-supplemented medium as follows. A base medium containing 7H9 powder (4.7g/L), NaCl (100mM), tyloxapol (0.05%), and fatty acid-free BSA (0.5g/L) was prepared and heated to 37°C, and a cholesterol stock solution was prepared by dissolving cholesterol in a 1:1 mixture of ethanol and tyloxapol and heated to 80°C for 30 minutes. This mixture was then added to pre-warmed base medium at a final concentration of 0.2 mM. Mtb were grown in standard medium to mid-log phase and were sub-cultured once prior to acclimation. Once acclimated to cholesterol medium, cultures were sub-cultured once into fresh medium and grown to mid-log phase prior to drug treatment assays.

Growth rate inhibition (GR) metrics calculation

GR values were derived using the following equation[31]:

$$\mathrm{GR(c)} = 2^{\frac{log_2(\frac{x(c)}{x_0})}{log_2(\frac{x_{ctrl}}{x_0})}} - 1$$

Where x(c) is the $OD_{600}$ value of a drug-treated sample at concentration c at a specific time point, $x_{ctrl}$ is the measurement of the untreated control at the same time point, and $x_0$ is the $OD_{600}$ value measured immediately prior to drug exposure (time point 0). GR values quantify the relative growth rate of treated cells compared with untreated cells.

GR was calculated for each drug concentration in the dose–response curve, and a three-parameter Hill function was fit to the data:

$$\mathrm{GR(c)} = \mathrm{GR_{inf}} + \frac{1 - \mathrm{GR_{inf}}}{1 + (\frac{c}{\mathrm{EC}_{50}^{GR}})^{h_{\mathrm{GR}}}}$$

Where $\mathrm{GR_{inf}}$ denotes the maximal inhibitory effect, $\mathrm{EC}_{50}^{GR}$ is the concentration at half-maximal growth rate inhibition, $h_{\mathrm{GR}}$ is the Hill slope, and c is the concentration of the drug

supplied. $\mathrm{GR_{max}}$ is the maximum effect of a drug at its highest tested concentration and ranges from -1 to 1. A negative value corresponds to a cytotoxic response, a value of 0 corresponds to a cytostatic response, and a positive value corresponds to partial growth inhibition.

By definition, GRmax values range from -1 (complete cytotoxicity) to 1 (no inhibition). In cholesterol, where the doubling times for $x_{ctrl}$ can exceed 90h for some strains, the denominator in the GR equation, $log_2(\frac{x_{ctrl}}{x_0})$ becomes small, making the metric sensitive to minor sources of error such as background noise. Additionally, cholesterol often precipitates over the course of the 12-day incubation step and can increase apparent $OD_{600}$ readings. These effects can occasionally produce computed GR values slightly below -1, which are not biologically possible (as they imply more than 100% loss of cells) and therefore represent numerical artifacts rather than true phenotypes. We therefore bounded all values less than -1 to -1, preserving the intended scale of GR and preventing spurious extreme values from influencing downstream analyses.

Drug interaction quantification

For drug combinations, drug interactions were quantified using Loewe additivity as the null model to compute fractional inhibitory concentration (FIC) values. For each drug combination, the expected IC value was calculated by summing the individual IC values of each constituent drug. The FIC value was then computed as the ratio of the experimentally observed IC value of the drug combination to the expected IC value. FIC values were $\log_2$-transformed to center synergy and antagonism scores at 0. A negative $\log_2$FIC value indicates synergy, a positive $\log_2$FIC value indicates antagonism, and a $\log_2$FIC value at zero indicates additivity. FIC values were calculated at $IC_{50}$ ($FIC_{50}$).

## Supplementary Tables and Figures

| Drug | Abbreviation | Mechanism of action |
|---|---|---|
| Bedaquiline | BDQ | Respiration |
| Telacebec | Q203 | Respiration |
| Pyrazinamide | PZA | Respiration or Multi–effect |
| Isoniazid | INH | Cell wall |
| SQ109 | SQ109 | Cell wall |
| TBA–7371 | TBA | Cell wall |
| Moxifloxacin | MOX | DNA |
| Rifampicin | RIF | RNA |
| Linezolid | LIN | Protein |
| Pretomanid | PRE | Multi–effect |

**Supplementary Table 1** Names, abbreviations, and mechanisms of action of the ten antibiotics used in this study.

For all ten antibiotics, the commercial name, the abbreviation used in this study, and its mechanism of action are listed in the left-most, middle, and right-most columns, respectively.

| Single drugs | Corresponding drug pairs |
|---|---|
| BDQ | BDQ + PRE |
| | BDQ + SQ109 |
| | BDQ + LIN |
| | BDQ + INH |
| | BDQ + MOX |
| | BDQ + RIF |
| | BDQ + Q203 |
| | BDQ + TBA |
| | BDQ + PZA |
| PRE | PRE + BDQ |
| | PRE + SQ109 |
| | PRE + LIN |
| | PRE + INH |
| | PRE + MOX |
| | PRE + RIF |
| | PRE + Q203 |
| | PRE + TBA |
| | PRE + PZA |
| SQ109 | SQ109 + BDQ |
| | SQ109 + PRE |
| | SQ109 + LIN |
| | SQ109 + INH |
| | SQ109 + MOX |
| | SQ109 + RIF |
| | SQ109 + Q203 |
| | SQ109 + TBA |
| | SQ109 + PZA |
| LIN | LIN + BDQ |
| | LIN + PRE |
| | LIN + SQ109 |

| Single drugs | Corresponding drug pairs |
|---|---|
| | LIN + INH |
| | LIN + MOX |
| | LIN + RIF |
| | LIN + Q203 |
| | LIN + TBA |
| | LIN + PZA |
| INH | INH + BDQ |
| | INH + PRE |
| | INH + SQ109 |
| | INH + LIN |
| | INH + MOX |
| | INH + RIF |
| | INH + Q203 |
| | INH + TBA |
| | INH + PZA |
| MOX | MOX + BDQ |
| | MOX + PRE |
| | MOX + SQ109 |
| | MOX + LIN |
| | MOX + INH |
| | MOX + RIF |
| | MOX + Q203 |
| | MOX + TBA |
| | MOX + PZA |
| RIF | RIF + BDQ |
| | RIF + PRE |
| | RIF + SQ109 |
| | RIF + LIN |
| | RIF + INH |
| | RIF + MOX |
| | RIF + Q203 |

| Single drugs | Corresponding drug pairs |
| --- | --- |
| | RIF + TBA |
| | RIF + PZA |
| Q203 | Q203 + BDQ |
| | Q203 + PRE |
| | Q203 + SQ109 |
| | Q203 + LIN |
| | Q203 + INH |
| | Q203 + MOX |
| | Q203 + RIF |
| | Q203 + TBA |
| | Q203 + PZA |
| TBA | TBA + BDQ |
| | TBA + PRE |
| | TBA + SQ109 |
| | TBA + LIN |
| | TBA + INH |
| | TBA + MOX |
| | TBA + RIF |
| | TBA + Q203 |
| | TBA + PZA |
| PZA | PZA + BDQ |
| | PZA + PRE |
| | PZA + SQ109 |
| | PZA + LIN |
| | PZA + INH |
| | PZA + MOX |
| | PZA + RIF |
| | PZA + Q203 |
| | PZA + TBA |

**Supplementary Table 2** Corresponding drug pairs of each individual drug used in this study.

All ten antibiotics and all nine corresponding drug pairs of each individual antibiotic are listed on the left-most and right-most columns, respectively.

| Three-way combinations |
| --- |
| BDQ + PRE + LIN |
| BDQ + PRE + INH |
| BDQ + PRE + MOX |
| BDQ + PRE + RIF |
| BDQ + LIN + INH |
| BDQ + LIN + MOX |
| BDQ + LIN + RIF |
| BDQ + INH + MOX |
| BDQ + INH + RIF |
| BDQ + MOX + RIF |
| PRE + LIN + INH |
| PRE + LIN + MOX |
| PRE + LIN + RIF |
| PRE + INH + MOX |
| PRE + INH + RIF |
| PRE + MOX + RIF |
| LIN + INH + MOX |
| LIN + INH + RIF |
| LIN + MOX + RIF |
| INH + MOX + RIF |

**Supplementary Table 3** All three-way combinations tested in this study.

| Strain | SRA accession number |
|---|---|
| 044 | SRR5073757 |
| 070 | SRR5074078 |
| 082 | SRR5073981 |
| 084 | SRR5073991 |
| 139 | SRR5073977 |
| 185 | SRR5073510 |
| 245 | SRR5067510 |
| 355 | SRR5067598 |
| 358 | SRR5067503 |
| 414 | SRR5067585 |
| 478 | SRR5065461 |
| 545 | SRR5065319 |
| 617 | SRR5065320 |
| Erdman | SRR24083712 |

**Supplementary Table 4** Identification and accession numbers of the 14 strains used in this study.

For all 14 strains used in this study, the identification name and Sequence Read Archive (SRA) accession numbers are listed in the left and right columns, respectively.

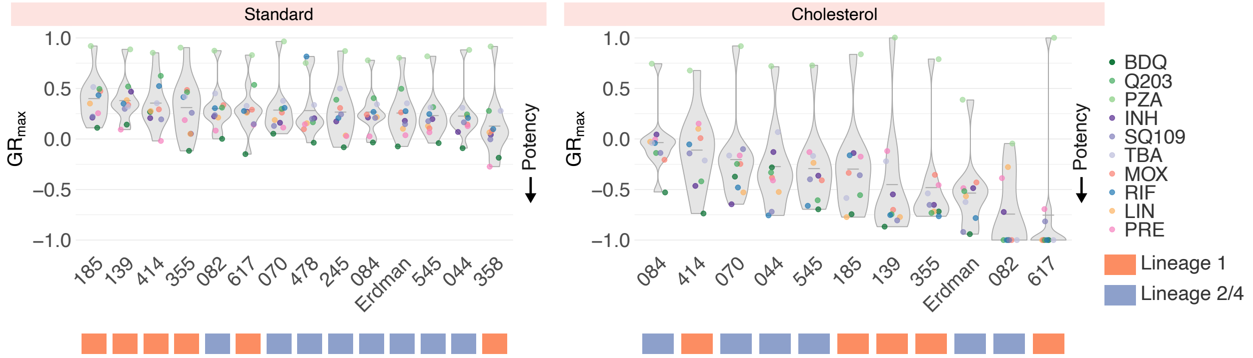


**Supplementary Figure 1** Susceptibility profiles of 14 Mtb strains across ten antibiotic treatments in standard (left) and 11 strains in cholesterol (right). Strains are ordered from least drug-susceptible to most drug-susceptible (left to right, respectively). Each point represents the median $GR_{max}$ value across six biological replicates in standard and three biological replicates in cholesterol.

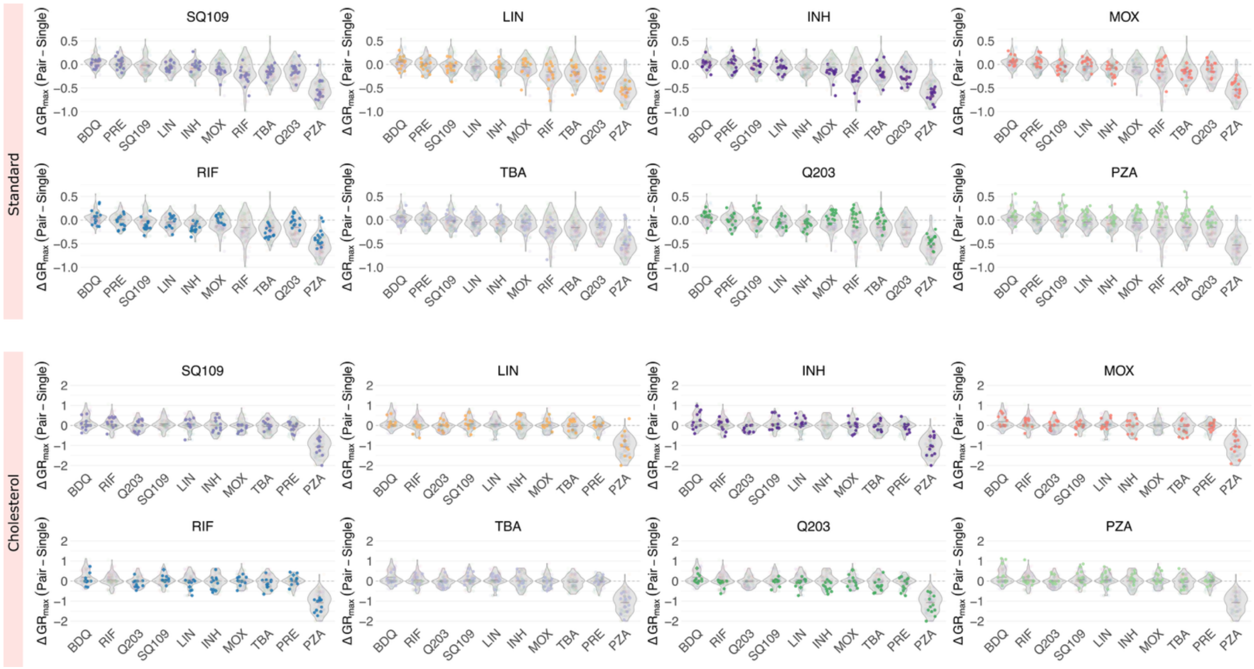


**Supplementary Figure 2** Comparison of $GR_{max}$ values between single drugs and drug pairs.

In both standard (top) and cholesterol (bottom) conditions, each of the eight panels represents the change in $GR_{max}$ between a single drug and its 9 corresponding pairs. $\Delta GR_{max}$ is calculated by subtracting the $GR_{max}$ value of the single drug from that of a corresponding drug pair. For each single drug, all nine corresponding drug pairs were tested in fourteen clinical isolates in standard and eleven clinical isolates in cholesterol. Each point denotes a $GR_{max}$ value of a drug pair for an individual isolate and represents the median $GR_{max}$ value across three biological replicates. A negative $\Delta GR_{max}$ value indicates an improvement in $GR_{max}$ when a single drug is combined with another drug.

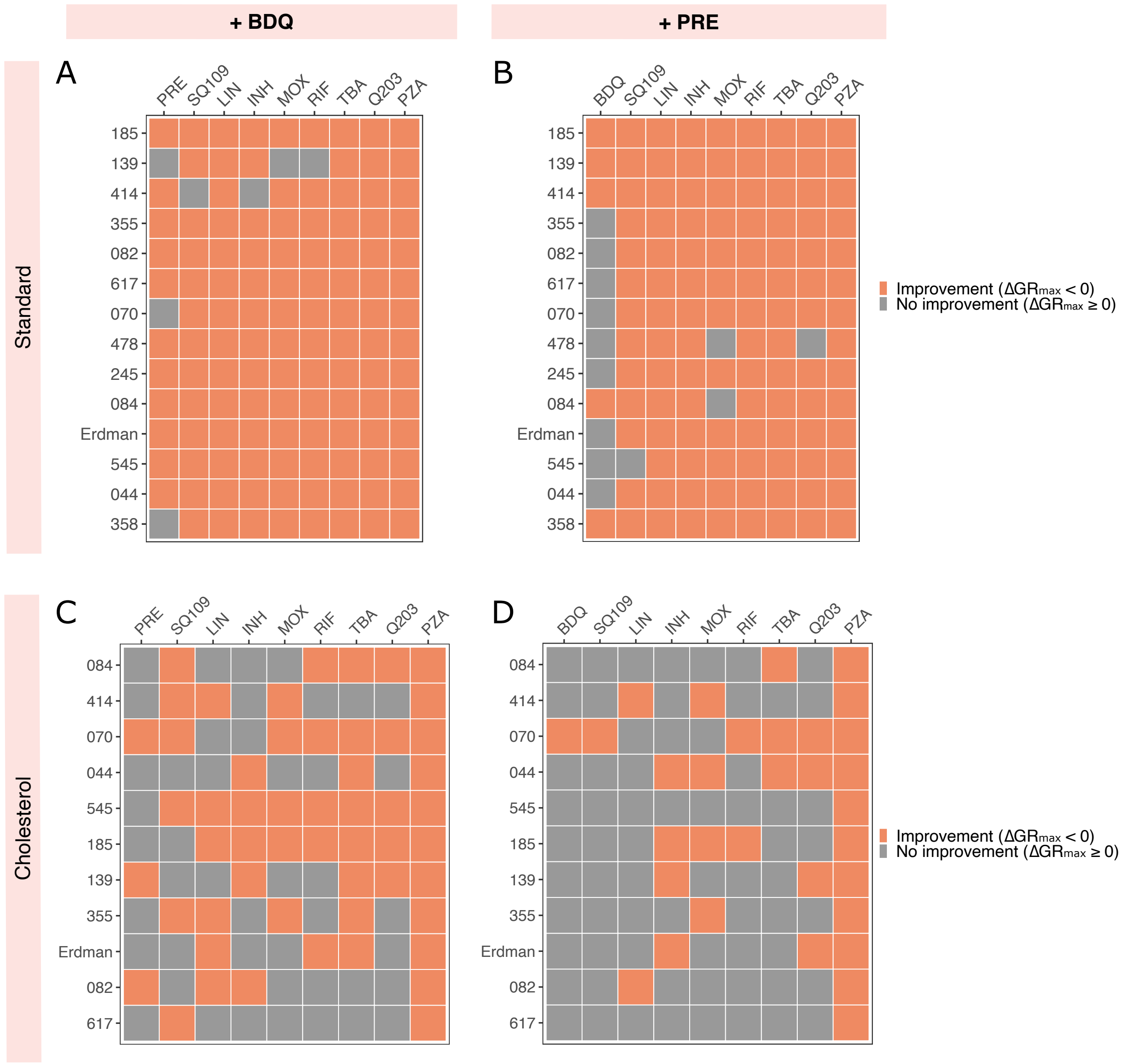


**Supplementary Figure 3** $GR_{max}$ improvement in drug pairs containing BDQ (left) or PRE (right) in standard (A-B) and cholesterol (C-D). Strain names are listed on the left-most column, and drug names are listed on the top row of each heat map. Orange tiles indicate an improvement in $GR_{max}$ when a single drug is combined with either BDQ or PRE ($\Delta GR_{max} < 0$; Figure 2A), and gray tiles indicate no improvement ($\Delta GR_{max} \geq 0$).

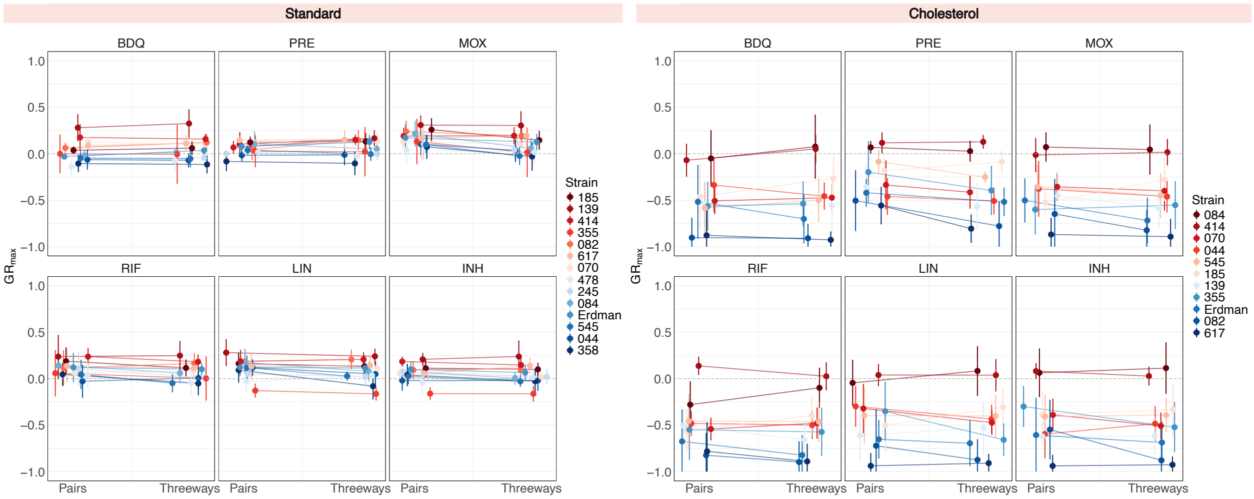


**Supplementary Figure 4** Comparison of $GR_{max}$ values between drug pairs and three-way combinations.

In both standard (left) and cholesterol (right) conditions, each of the six panels represents a single drug, with the mean $GR_{max}$ value of its 5 corresponding drug pairs on the left side and the mean $GR_{max}$ value of its ten corresponding three-way combinations on the right side. Vertical bars represent the standard deviation of the mean $GR_{max}$ value. Each point corresponds to one of 14 clinical isolates in standard (left), or one of 11 clinical isolates in cholesterol (right).

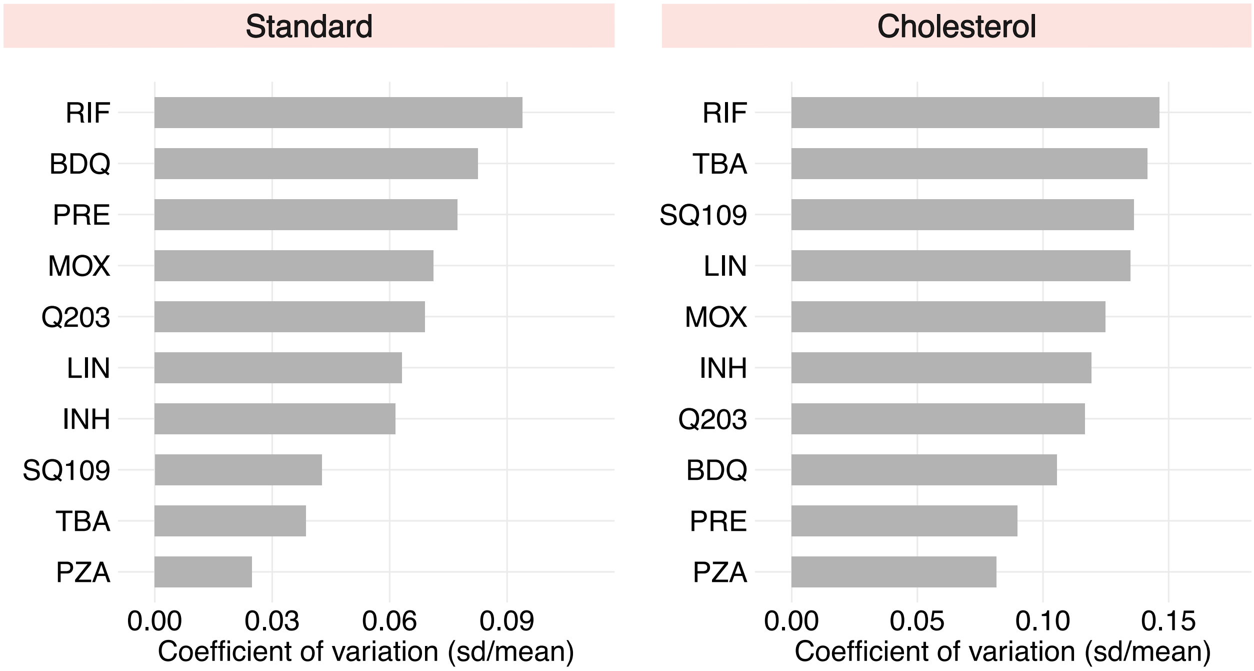


**Supplementary Figure 5** Coefficient of variation (CV) values of ten single drugs

In both standard (left) and cholesterol (right) conditions, the CV was calculated for each drug by dividing the standard deviation of the $GR_{max}$ values by the mean $GR_{max}$ values of 14 isolates in standard and 11 isolates in cholesterol. Drugs were rank ordered from largest to smallest CV (top to bottom, respectively), with larger values indicating greater variation in $GR_{max}$ between strains.

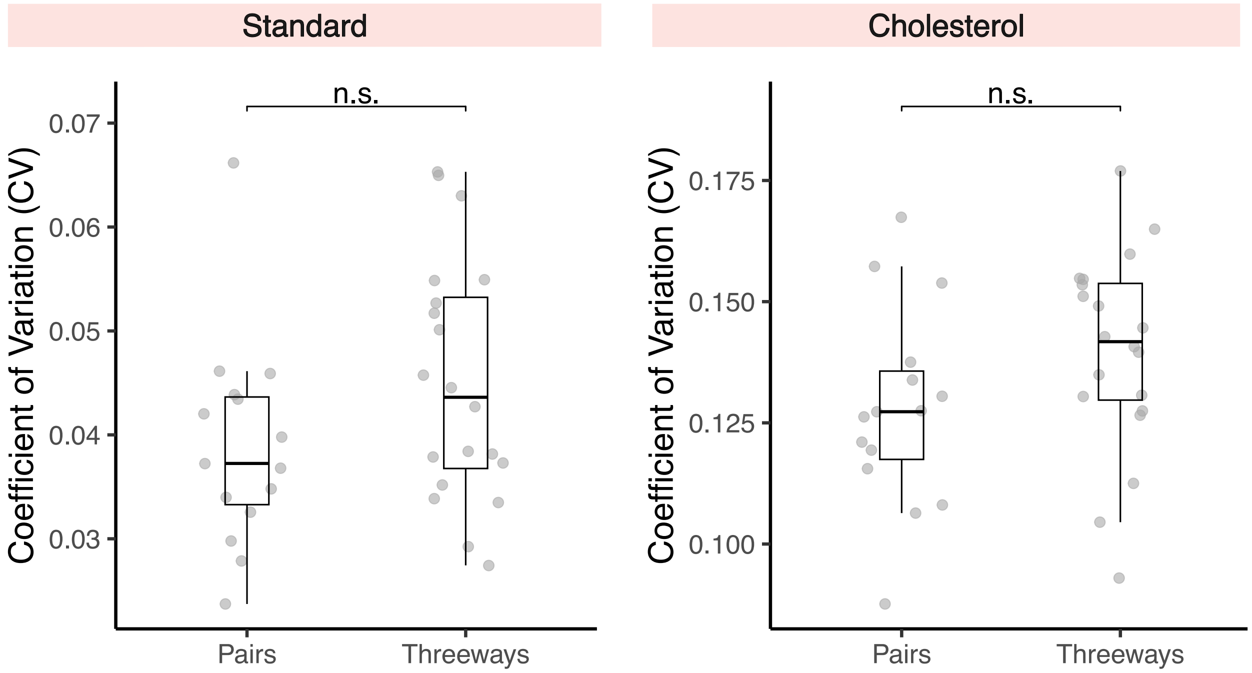


**Supplementary Figure 6** Comparison of mean coefficient of variation (CV) between drug pairs and three-way combinations.

In both standard (left) and cholesterol (right) conditions, the CV of each drug pair (total of 15) and three-way combinations (total of 20) was calculated by dividing the standard deviation of the $GR_{max}$ values by the mean $GR_{max}$ values of 14 isolates in standard and 11 isolates in cholesterol. A Student's t-test was performed to determine whether the difference between the mean CV of drug pairs and the mean CV of three-way combinations is statistically significant.
